# Supplementary material for: Effect of Vibrio-Derived Extracellular Protease vEP-45 on the Blood Complement System
Source: Biology (Basel). 2021 Aug 18;10(8):798. doi: 10.3390/biology10080798 (PMC8389632; doi:10.3390/biology10080798)
Supplement: Supplementary file 1 [file biology-10-00798-s001.zip › biology-1317701-supplementary.pdf]

Figure S1

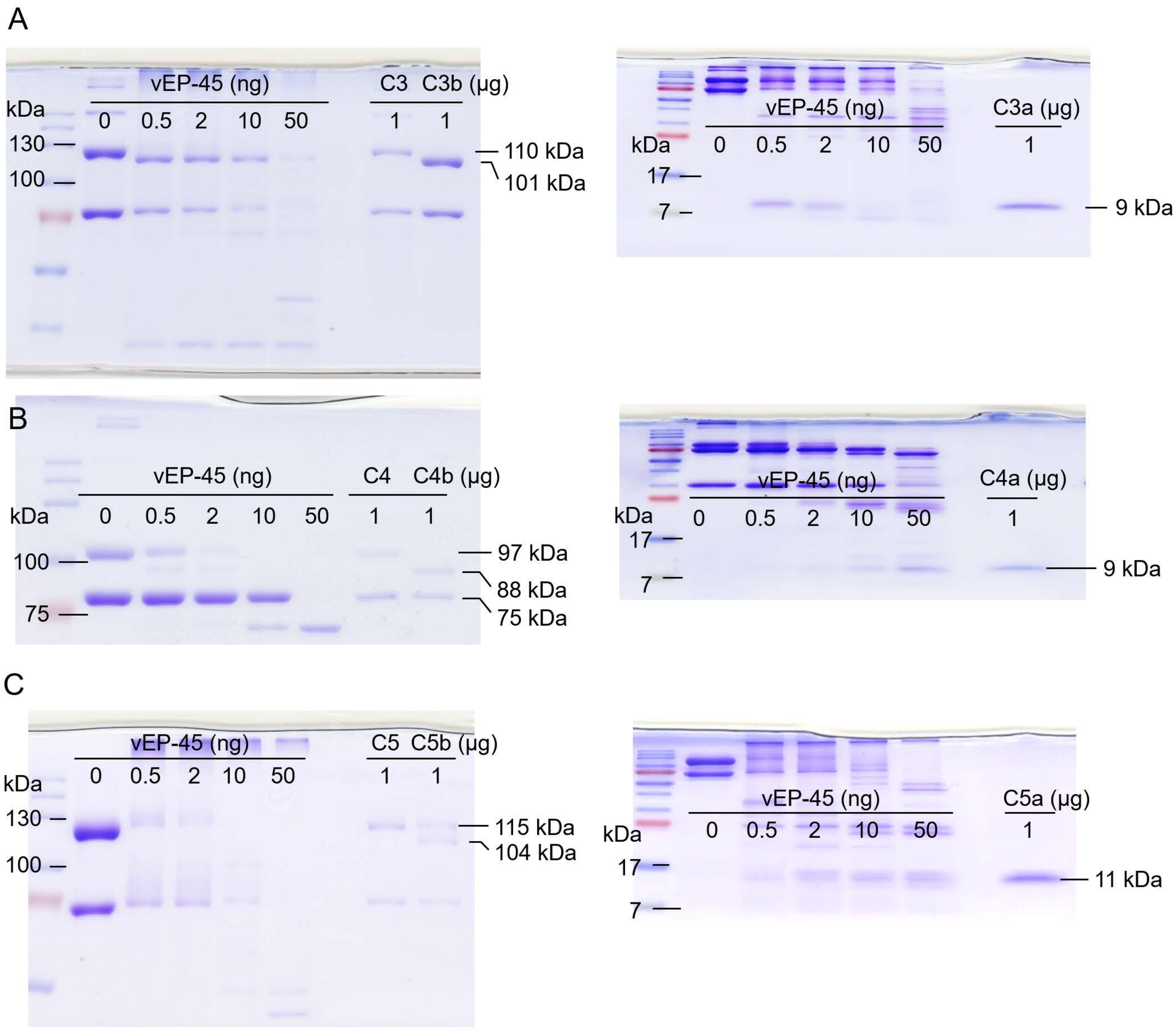

Figure S1. Cleavage of human complement proteins (C3, C4, and C5) by vEP-45 as determined by SDS-PAGE.

Figure S2

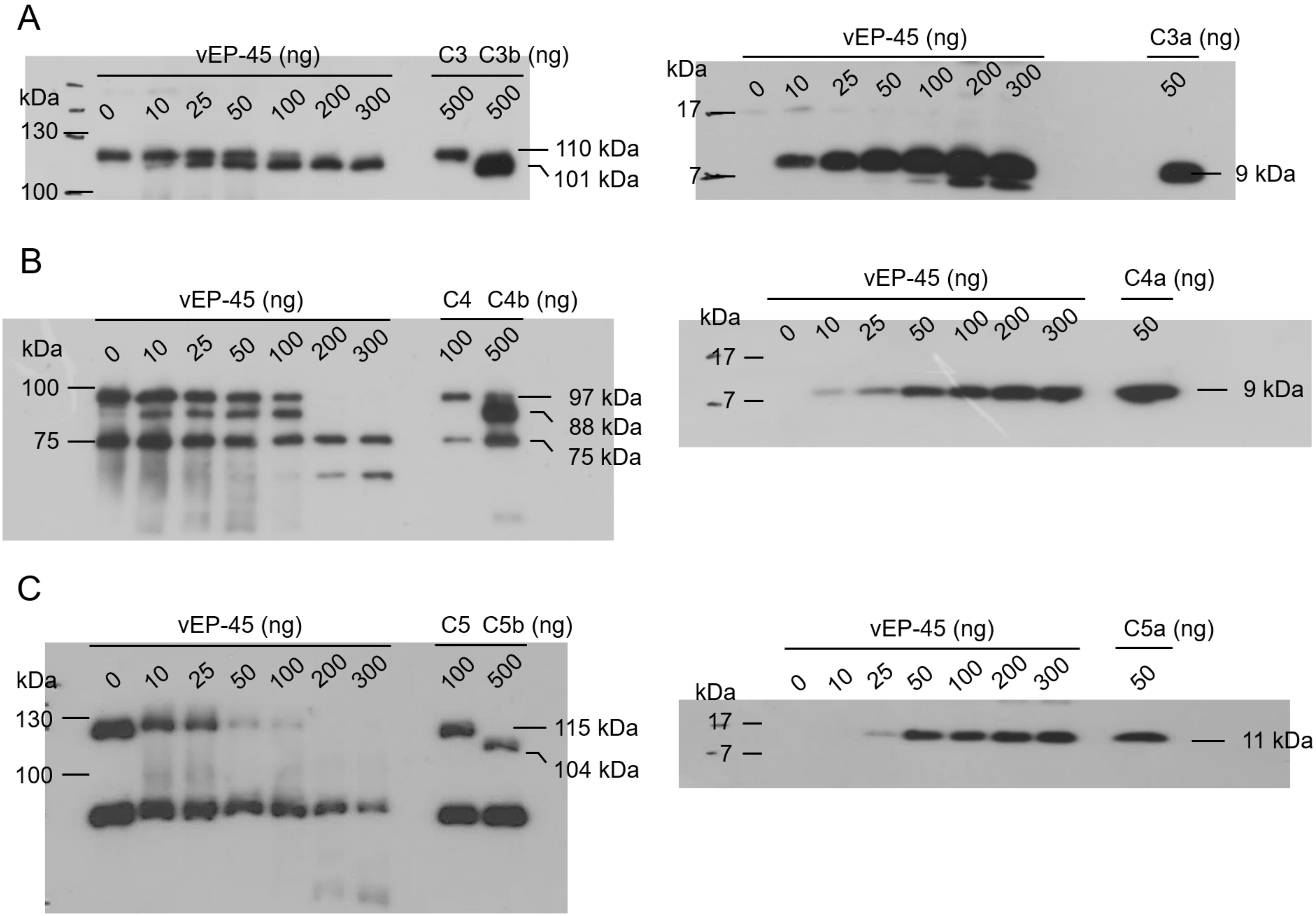

Figure S2. Cleavage of human complement proteins by vEP-45 as detected with Western blotting.

Figure S3

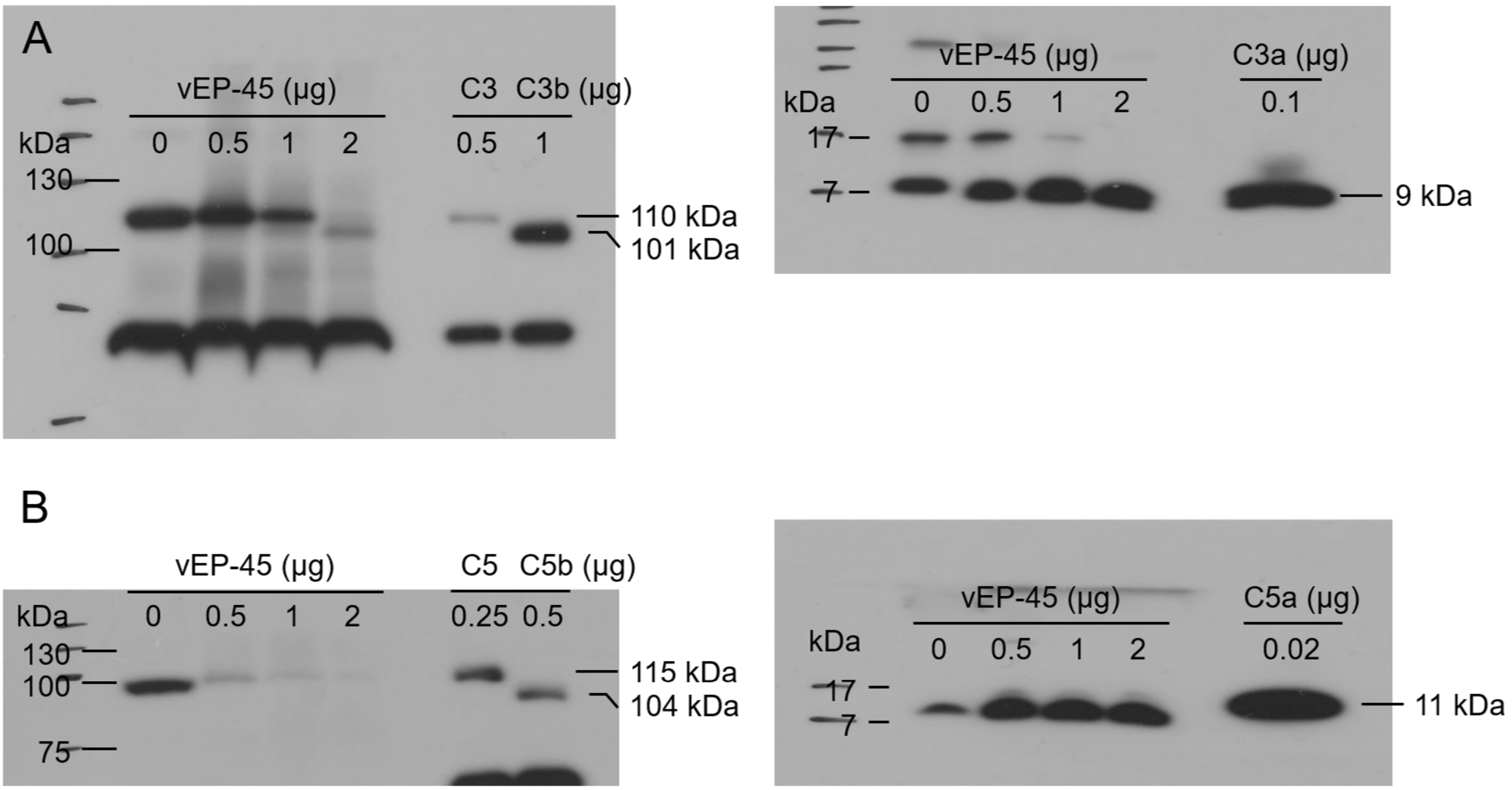

Figure S3. Cleavage of C3 and C5 by vEP-45 in human plasma.

Figure S4

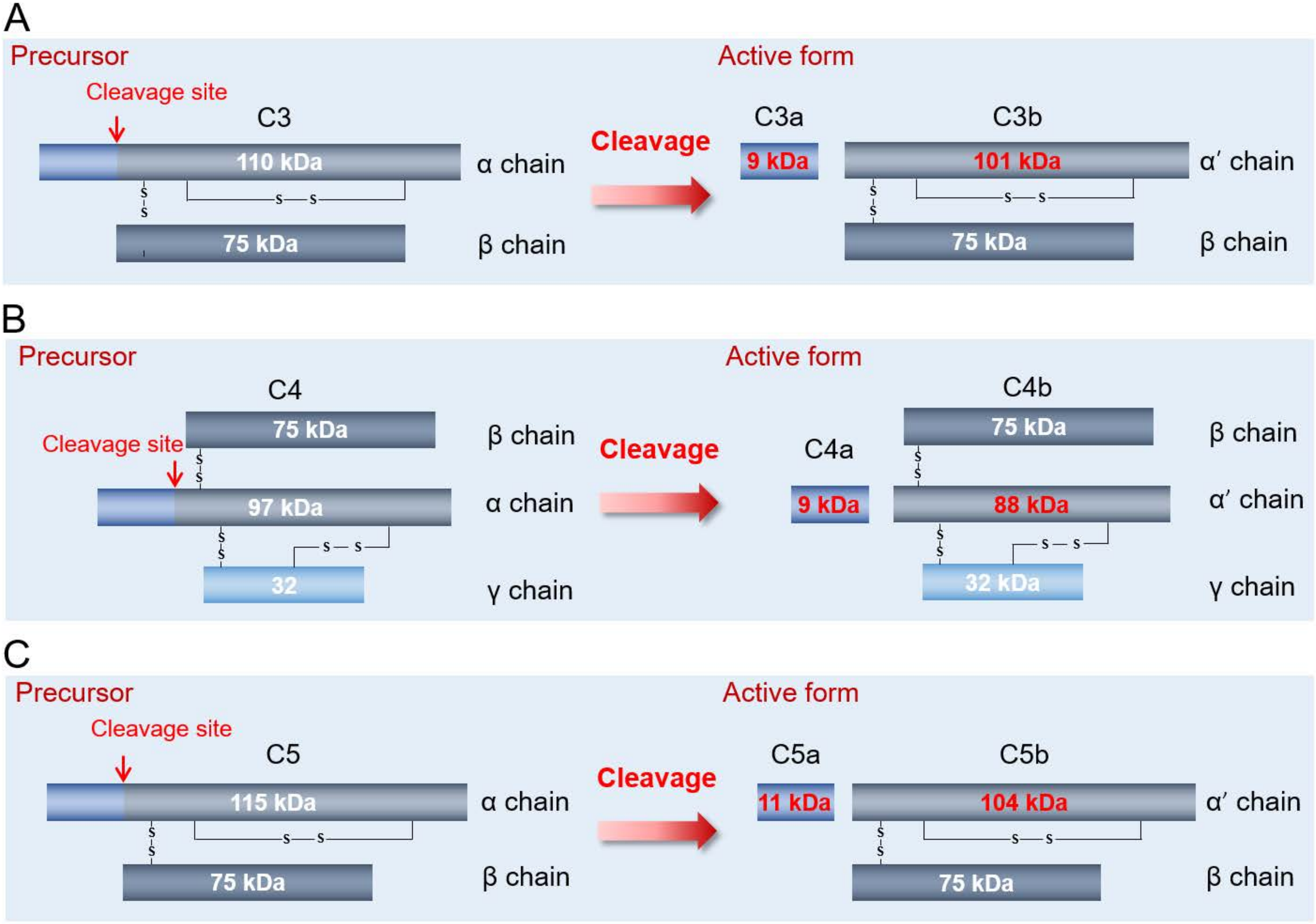

Figure S4. Schematic structure of C3 (A), C4 (B), and C5 (C).
